# Validation and Assessment of Three Methods to Estimate 24-h Urinary Sodium Excretion from Spot Urine Samples in Chinese Adults

**DOI:** 10.1371/journal.pone.0149655

**Published:** 2016-02-19

**Authors:** Yaguang Peng, Wei Li, Yang Wang, Hui Chen, Jian Bo, Xingyu Wang, Lisheng Liu

**Affiliations:** 1 State Key Laboratory of Cardiovascular Disease, Fuwai Hospital, National Center for Cardiovascular Disease, Chinese Academy of Medical Sciences and Peking Union Medical College, Beijing, China; 2 Beijing Hypertension League Institute, Beijing, China; Shanghai Institute of Hypertension, CHINA

## Abstract

24-h urinary sodium excretion is the gold standard for evaluating dietary sodium intake, but it is often not feasible in large epidemiological studies due to high participant burden and cost. Three methods—Kawasaki, INTERSALT, and Tanaka—have been proposed to estimate 24-h urinary sodium excretion from a spot urine sample, but these methods have not been validated in the general Chinese population. This aim of this study was to assess the validity of three methods for estimating 24-h urinary sodium excretion using spot urine samples against measured 24-h urinary sodium excretion in a Chinese sample population. Data are from a substudy of the Prospective Urban Rural Epidemiology (PURE) study that enrolled 120 participants aged 35 to 70 years and collected their morning fasting urine and 24-h urine specimens. Bias calculations (estimated values minus measured values) and Bland-Altman plots were used to assess the validity of the three estimation methods. 116 participants were included in the final analysis. Mean bias for the Kawasaki method was -740 mg/day (95% CI: -1219, 262 mg/day), and was the lowest among the three methods. Mean bias for the Tanaka method was -2305 mg/day (95% CI: -2735, 1875 mg/day). Mean bias for the INTERSALT method was -2797 mg/day (95% CI: -3245, 2349 mg/day), and was the highest of the three methods. Bland-Altman plots indicated that all three methods underestimated 24-h urinary sodium excretion. The Kawasaki, INTERSALT and Tanaka methods for estimation of 24-h urinary sodium excretion using spot urines all underestimated true 24-h urinary sodium excretion in this sample of Chinese adults. Among the three methods, the Kawasaki method was least biased, but was still relatively inaccurate. A more accurate method is needed to estimate the 24-h urinary sodium excretion from spot urine for assessment of dietary sodium intake in China.

## Introduction

Dietary sodium intake is positively associated with high blood pressure [1]. Several studies have supported this association including animal studies [2], randomized controlled trials [3, 4], observational studies [5–7], and meta-analyses [8, 9]. Dietary sodium intake has also been shown to increase the risk of coronary heart disease and stroke [10, 11]. While several guidelines include recommendations regarding dietary sodium intake [12–14], the scientific evidence to support these recommendations is mixed. Further epidemiological research in large, diverse, population-based studies is needed.

Currently, two approaches are typically used to assess dietary sodium intake: questionnaires and urinary sodium excretion. While questionnaires are inexpensive and have a relatively low participant burden, they may not accurately capture true dietary sodium intake [15]. 24-h urinary sodium excretion is therefore the preferred method and is considered the gold standard for assessing dietary sodium intake. However, collecting 24-h urine samples is time-intensive, expensive, and has a high participant burden, so methods for estimating 24-h urinary sodium excretion from spot urine samples have been developed. These include the Kawasaki method [16], the INTERSALT method [17], and the Tanaka method [18], which were mostly common used at the present. The validity of these estimation methods in the Chinese population has not been assessed. The objective of this study was to assess the validity of these three estimation methods against the gold standard 24-h urinary sodium excretion in a sample of Chinese adults.

## Methods

### Design and study participants

Data are from a subsample of the Prospective Urban Rural Epidemiology (PURE) study, which was an international multi-center prospective study [19, 20]. A total of 120 participants (60 rural and 60 urban) aged 35 to 70 years from the ongoing PURE study in Shanxi Province, China, were enrolled in the substudy through randomly sample upon attending either their 3-year or 6-year follow-up visit. The substudy was approved by the Ethics Committee of Fuwai Hospital and all participants provided written informed consent.

Exclusion criteria for the substudy were as follows: 1) use any diuretic drug; 2) pregnant or currently breastfeeding and 3) food restrictions due to chronic illness (e.g. kidney disease, cancer, HIV, renal or heart failure).

A history of diabetes and stroke, based on self-reported, were obtained from individual standardized questionnaire. Hypertension was defined by self-reported or a measured blood pressure level ≥140/90 mmHg at physical examination. Individual prescription medication information was recorded.

### Procedure

Participants were instructed to collect their urine over a 24-h period. They recorded the start and finish times of their collection, time of any missed urine passes, physical activities, any medications used during the collection, and any use of water softeners. Participants also took a morning fasting urine sample on next day morning, at the end of the 24-h collection. Thirty days after the first 24-h urine collection, the same 120 participants repeated the 24-h and morning fasting urine sample collections to estimate reproducibility. Within 12 hours of completion of each collection, the participants were asked to drop off the urine containers at their site. During the first visit, participants also completed standardized and validated questionnaires regarding medication use and lifestyle practices (diet, physical activity, and smoking), and blood pressure, weight, height, and waist circumference were measured by researchers.

### Specimen collection and analysis

Participants were provided with containers to collect the urine sample including a plastic bottle (300 mL) for the morning fasting urine and a plastic bucket (4 L) for the 24-h urine sample, as well as detailed written instructions on how to collect the sample. The 24-h urine was voided into the plastic bucket without omission. The next morning the morning fasting urine was collected in a separate bottle. Upon receipt of the samples at the study site, 2 mL of the morning fasting urine was stored in a plastic eppendorf tube and what remained was combined with the 24-h urine sample. The 24-h urine sample was then mixed with a glass stick and 2 mL was stored in a plastic eppendorf tube. Samples were stored at 4°C for 24-h and frozen at -20°C within 7 days and dispatched to the study center lab in Beijing where the analysis of sodium (Na^+^), potassium (K^+^), and creatinine (Cr) was carried out. Na^+^, K^+^ were examined by emission flame photometry and Cr by the Jaffe method.

### Statistical analysis

Participants with incomplete urine collections or missing data were excluded from this analysis (n = 4, 3.3%). Continuous variables are presented as mean±standard deviation (SD) and categorical variables are presented as % (n).

The measured (“true”) 24-h urinary sodium excretion was calculated using the following equation:

$$24\text{-}h\;urinary\;sodium\;excretion\;\left(mg/day\right)=concentration\;of\;24\text{-}h\;urinary\;sodium\;excretion\;\left(mmol/L\right)\times 24\text{-}h\;urine\;volume\;\left(L/day\right)\times molecular\;weight\;of\;Na^{+}\;\left(23\;mg/mmol\right)$$

Three different methods, for estimating 24-h urinary sodium excretion using the morning fasting urine sample: Kawasaki, INTERSALT and Tanaka methods were listed in **Table 1**.

**Table 1:** Three methods to estimated 24-hour urinary sodium excretion.

Method	Urine Sample	Formula to Estimate 24-h urinary sodium excretion (mg/day)
Kawasaki	Second morning urine	23×16.3×(Na_spot_/Cr_spot_×PrUCr_24h_)^0.5^
		PrUCr_24h_ = 15.12×Weight+7.39×Height-12.63×Age-79.9 (Male)
		PrUCr_24h_ = 8.58×Weight+5.09×Height-4.72×Age-74.95 (Female)
INTERSALT	Casual Spot Urine	23×((25.46+0.46×Na_spot_)-2.75×Cr_spot_-0.13×K_spot_+4.10×BMI+0.26×Age) (Male)
		23×((5.07+0.34×Na_spot_)-2.16×Cr_spot_-0.09×K_spot_+2.39×BMI+2.35×Age-0.03×Age^2^) (Female)
Tanaka	Casual Spot Urine	23×21.98×(Na_spot_/Cr_spot_×PrUCr_24h_)^0.392^
		PrUCr_24h_ = 14.89×Weight+16.14×Height-2.04×Age-2244.45

Note: Predicted 24-h urinary creatinine, PrUCr_24h_; Spot urinary sodium, Na_spot_; Spot urinary potassium, K_spot_; Spot urinary creatinine, Cr_spot_; The units of concentration of Na_spot_, K_spot_, Cr_spot_ were all mmol/L, and the unit of PrUCr_24h_ was mg/day. Weight and Height were kg and cm. The molecule weight of Na was 23 mg/mmol.

The validity of the three estimation methods relative to the true measured 24-h urinary sodium excretion was evaluated using correlation analysis including linear correlation and interclass correlation coefficients (ICCs), and visualized using scatter plots. The estimated values of 24-h urinary sodium excretion were calculated for each of the three formulas (Table 1). The differences were computed by estimated values of 24-h urinary sodium excretion minus the measured value. Bland-Altman plots were also used to evaluate systematic bias [21]. A value of P<0.05 was considered statistically significant. All analyses were performed using SPSS 20.0 (SPSS & IBM, Inc, Chicago, Illinois, USA).

## Results

A total of 116 participants were included in the final analysis. The characteristics of the participants are presented in **Table 2**.

**Table 2:** Characteristic of participants (n = 116).

	Means / N (%)
Age (years)	53.16±8.09
Female	79 (68.1)
Weight (kg)	63.23±10.67
Height (cm)	159.85±8.44
Body mass index (kg/m^2^)	25.61±6.02
Systolic blood pressure (mmHg)	140.39±20.66
Diastolic blood pressure (mmHg)	87.65±11.77
Hypertension	66 (56.9)
Diabetes (Self-reported)	7 (6.0)
Stroke (Self-reported)	3 (2.6)
**Morning fasting urine**	
Na+ concentration (mmol/L)	174.24±70.60
K+ concentration (mmol/L)	59.20±31.14
Creatinine concentration (mmol/L)	9.97±6.28
**24-hour urine**	
24-h Na+ concentration (mmol/L)	157.93±50.06
24-h K+ concentration (mmol/L)	27.59±11.69
24-h urine volume (mL)	1869.05±817.14

Values are mean±SD or n (%).

Mean 24-h urine volume was 1869.1 mL. The differences between estimated and measured 24-h urinary sodium excretion are presented in **Table 3**. The mean difference for the Kawasaki method was -740 mg/day (95% CI: -1219, 262 mg/day), and was the smallest difference of the three estimation methods. The largest difference was from the INTERSALT method: -2797 mg/day (95% CI: -3245, 2349 mg/day).

**Table 3:** Validity of three methods of estimation versus measured 24-h urinary sodium excretion in a sample of Chinese adults (n = 116).

	Measured	Kawasaki method	INTERSALT method	Tanaka method
**Mean** (mg/day)				
All	6343.02±2470.52	5602.53±1471.40	3545.63±873.86	4037.97±772.31
Women	6011.69±2481.83	5967.53±1451.18	3181.57±555.69	4026.70±760.94
Men	7050.45±2323.01	4823.21±1198.59	4322.95±926.55	4062.03±806.17
**Range** (mg/day)	561.66–15014.40	2151.57–9674.37	1994.14–6910.35	2251.53–6656.68
**Mean difference (95% CI)**	Reference	-740.49	-2797.39	-2305.05
(mg/day)		(-1218.73, -262.25)	(-3245.40, -2349.38)	(-2735.01,-1875.09)
**Intraclass correlation coefficient (95% CI)**	Reference	0.28 (0.00, 0.50)	0.21 (0.00, 0.45)	0.29 (0.00, 0.51)
**Pearson correlation coefficient**	Reference	0.187	0.187	0.293

Within gender subgroups, mean measured 24-h urinary sodium excretion was significantly higher among men compared to women (P = 0.03). With regard to the estimation methods, using the Kawasaki method, mean estimated 24-h urinary sodium excretion was significantly higher in women compared to men (P<0.001). Using the INTERSALT and Tanaka methods, mean estimated 24-h urinary sodium excretion was higher in men compared to women, but was only significantly different for the INTERSALT method (P<0.001) (**Table 3**).

**Fig 1:**
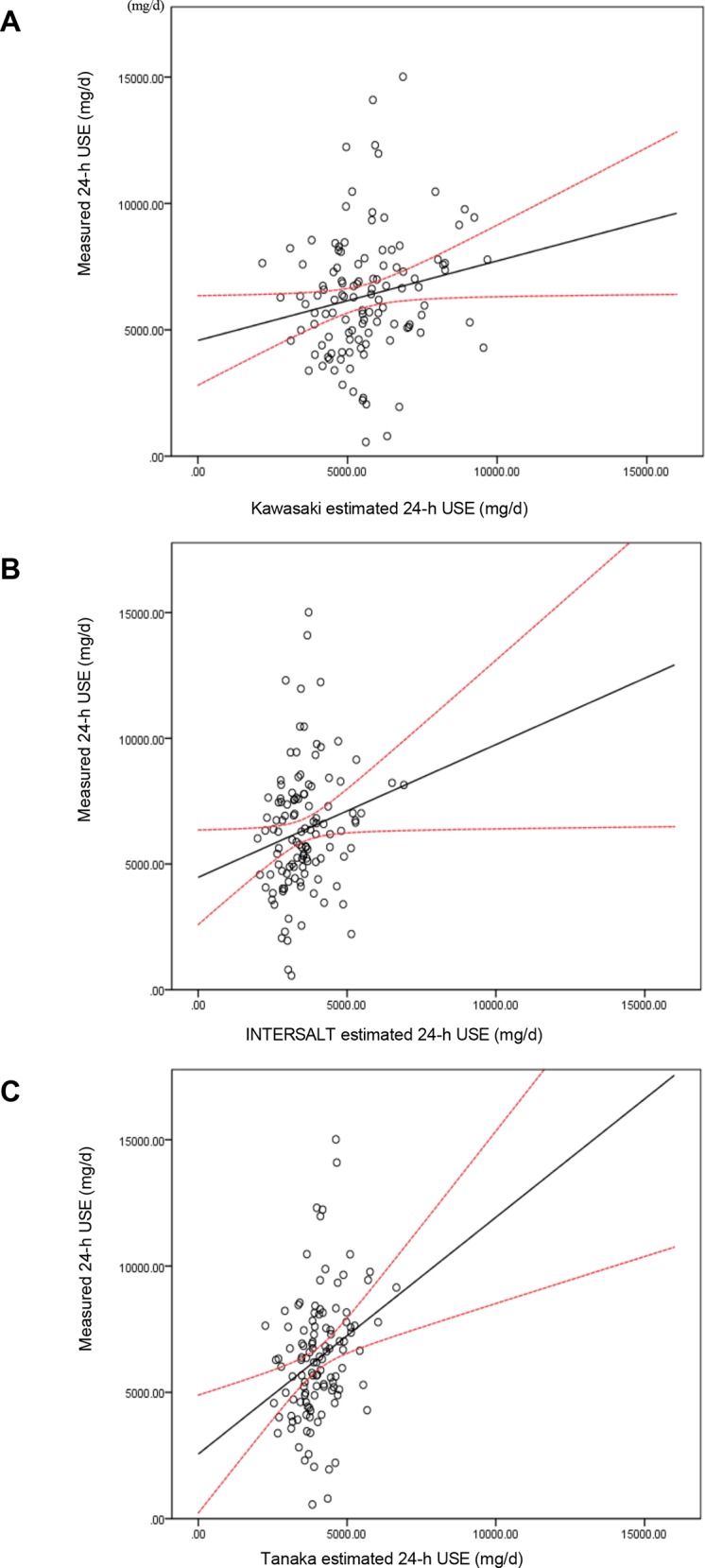
**Scatter plots measured 24-h urine sodium excretion (USE) vs. Kawasaki (A), INTERSALT (B), and Tanaka (C) method estimated 24-h USE (mg/d).** The hollow circles were scatter points of measured and estimated values. The real line was the linear regression line of the scatters in the plots. The dash lines were the 95% CI lines of predicted mean.

Pearson’s correlation coefficients between measured and estimated 24-h urinary sodium excretion were low: 0.19 for the Kawasaki and INTERSALT methods and 0.29 for the Tanaka method (**Table 3 and Fig 1**). ICCs were also low: 0.28 for the Kawasaki method, 0.29 for the Tanaka method, and 0.21 for the INTERSALT method.

**Fig 2:**
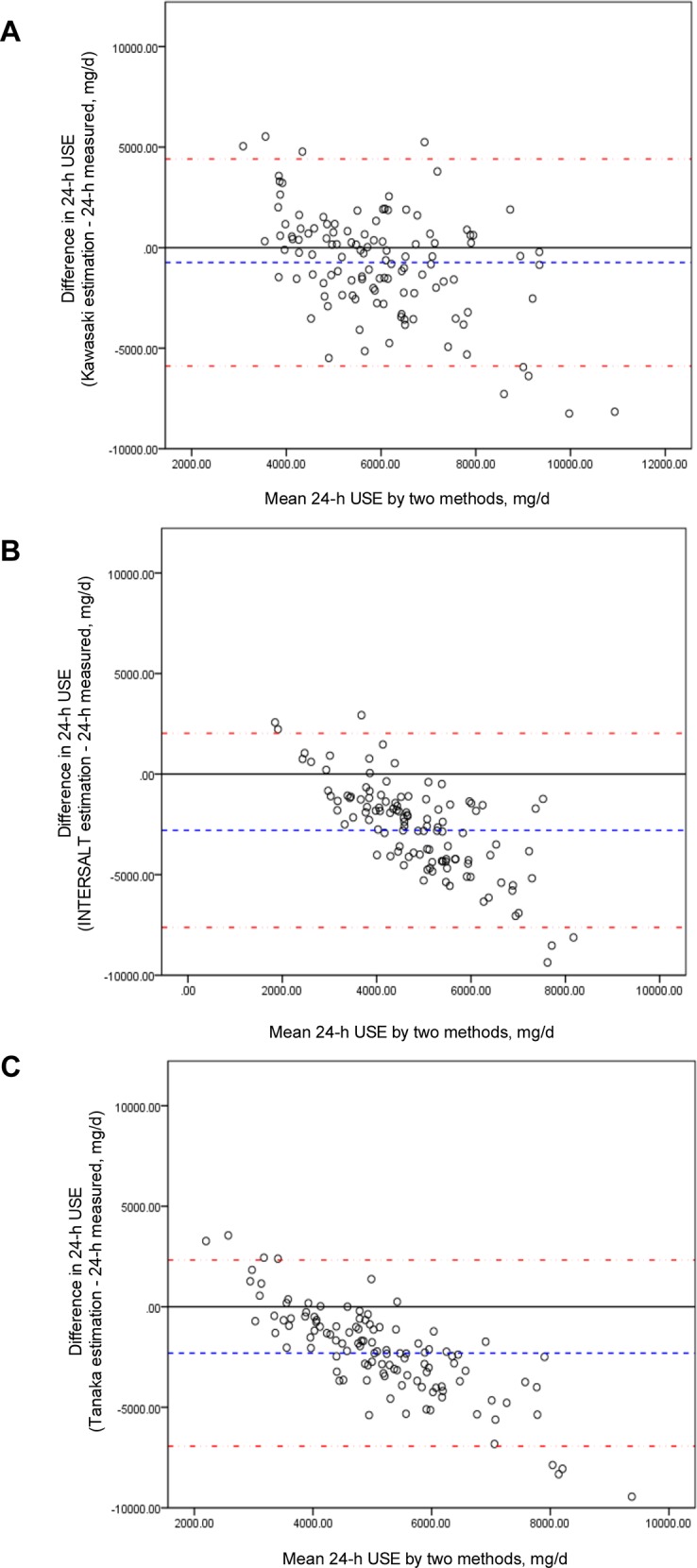
**Bland-Altman plots of measured 24-h urine sodium excretion (USE) vs. Kawasaki (A), INTERSALT (B), and Tanaka (C) method estimated 24-h USE (mg/d).** The difference between measured and estimated was all estimated values minus the measured values. The mid-dashed line was the mean difference or bias between measured and estimated values. The dash-point line represented the 95% limits of agreement of the mean difference ± 1.96 standard deviation.

The mean estimated 24-h urinary sodium excretion levels were consistently underestimated relative to the measured true value. The Kawasaki method using morning fasting urine performed a relative accuracy among the three methods: the least gap between the measured true 24-h urine sodium excretion in Bland-Altman plots (**Fig 2A**). The INTERSALT and Tanaka methods very consistently underestimated true 24-h urinary sodium excretion, with even poorer performance at higher levels of 24-h urinary sodium excretion (**Fig 2B and 2C**).

ICCs between the initial collection and the repeated collection for concentration of 24-h urine sodium and 24-h urine volume were 0.64 (95% CI: 0.48, 0.76) and 0.76 (95% CI: 0.66, 0.84), respectively, and for 24-hour urinary sodium excretion, 0.36 (95% CI: 0.06, 0.56). The correlation coefficients were all fair-to-moderate (for concentrations of morning fasting urine sodium, r = 0.50, P<0.001; for concentrations of 24-h urine sodium, r = 0.48, P<0.001; for concentrations of morning fasting urine Creatinine, r = 0.46, P<0.001; for 24-h urine volumes, r = 0.62, P<0.001).

## Discussion

We found that of three methods for estimating 24-h urinary sodium excretion using a morning fasting urine sample, the Kawasaki method provided the most accurate estimate compared to measured 24-h urinary sodium excretion in Chinese adults. However, all three methods underestimated 24-h urinary sodium excretion. Furthermore, we observed systematic bias for the INTERSALT and Tanaka methods (**Fig 2**), which showed a negative association between means and differences of estimation and measured values. To explore this inverse association, some subgroup analysis was taken. Depending on the quartile distribution of the measured 24-h urinary sodium excretion, over-estimation might be in lower sodium excretion group (lower percentile 25 of measured value) and underestimation in higher sodium excretion group (over percentile 75 of measured value) (**S1–S4 Tables**). This indicated if real 24-h urinary sodium excretion is high, the INTERSALT and the TANAKA methods might be particularly inaccurate evaluation for population with higher salt intakes.

The level of 24-h urinary sodium excretion was associated with the sodium concentration and volume of the 24-h urine, which might have a relation with drinking and sweating. Creatinine excretion in urine is considered to be fairly constant [22–25] and so it can be used to adjust for urine concentration. All three methods were adjusted by the concentration of spot creatinine excretion. With a consistency, the results of PURE study about validation on these three methods, showed the Kawasaki formula was the most valid and least biased method of estimating 24-h urinary sodium excretion from a single MFU and could applied in population studies [26]. However, another study in the US found that the Kawasaki method was the most biased and the INTERSALT method the least biased when using morning, afternoon, or evening spot urine samples, and the Tanaka method was the least biased when using overnight samples [27]. In our study, considering the convenience for the investigation, we just took the morning fasting urine as the spot urine to estimate 24-h urinary sodium excretion. Future studies in the Chinese population should expand to include additional spot urine samples.

A study in Britain and Italy found low agreement of these estimation methods with 24-h urine sodium excretion with ethnic differences [28]. Kawasaki produced a correlation coefficient of 0.73 with measured values in a Japanese sample using second morning voiding urines [16]. Tanaka adopted a similar estimation method but using random spot urines and reported a correlation coefficient of 0.54 in a Japanese population sample but a coefficient of 0.32 in a validation sample [18]. Similarly, in a US sample, correlation coefficients were moderate (0.4–0.6) for all prediction equations and times of spot urine collection. Bland-Altman plots indicated significant over- and under-estimation across low to high values of individual sodium intake [27]. Furthermore, poor reproducibility indicates that estimation methods perform inconsistently and might be affected by the concentration of Na^+^ and the volume of urine. Future research should explore methods to overcome these issues. Although the difference between the Kawasaki method and measured levels was relatively small compared to the other two estimation methods, the underestimation and the wide 95% confidence intervals limit the utility of this method to estimate 24-h urinary sodium excretion by using a morning fasting urine in the Chinese population. It is possible that the low accuracy of these estimation methods was related to the high levels of sodium intake in this sample. The mean 24-h urinary sodium excretion in this population was 6343 mg/day, or about 16 g/day of salt (1 mg Na^+^≈2.54 mg NaCl). From the results of INTERSALT study [6], among 32 countries, the highest sodium excretion was 242 mmol/day in China, or about 14 g/day of salt. Another study of 663 adults in Tianjin (Aged 20–64 years), a city in northern China, reported sodium excretion levels consistent with a dietary intake of about 15 g/day salt. The 2002 Chinese National Nutrition and Health Survey [29] reported that sodium intakes were 6007 mg/day (about 15.4 g/day salt) in urban and 6369 mg/day (about 15.6 g/day salt) in rural areas by estimation from food frequency questionnaire (FFQ) investigation. Another study of dietary intake [30], which enrolled 68,962 participants from 31 provinces in China, reported a mean level of salt intake of 12 g/day using24-h dietary recall method. A cross-sectional epidemiological study of 4680 persons aged 40 to 59 years from four countries (USA, China, UK, and Japan), the INTERMAP study [31], also found mean levels of 24-h urinary sodium excretion were 13.4 g/day in China, compared to 11.7 g/day in Japan, 9.6 g/day in USA and 8.6 g/day in UK. Further evaluation of the China INTERMAP site suggested that among three areas (Beijing, Shanxi, and Guangxi) the mean 24-h urinary sodium excretions were 6325 mg/day (about 16.1 g/day), 6164 mg/day (about 15.7 g/day) and 3197 mg/day (about 8.1 g/day), respectively [31]. The sample in the substudy presented here, from PURE, consisted of adults in rural and urban Shanxi province. Our observed mean level of 24-h urinary sodium excretion was similar to previously reported levels in China, and supported very high levels of salt intake in China: about 16 g/day. From Fig 2, the results of Bland-Altman plots indicated a negative association between means and differences of estimation and measured values. In other words, it seemed to indicate significant over-estimation at low sodium excretion, while under-estimation at high sodium excretion, which suggested that accuracy of estimated methods, especially in INTERSALT and Tanaka methods, indeed were weaker when 24-h urinary sodium excretions were higher or lower. That might indicated the formula would have bias for population with high salt intake relatively and might not fit for them. Clearly our future research is needed regarding accurate estimation methods for 24-h urinary sodium excretion using spot urine samples in the Chinese population.

There are several important limitations worth noting. This was a substudy of PURE and included only one province in China, thus limiting the generalizability of our results. Furthermore, women and elderly adults were more likely to participate, especially in rural areas where young men were often away from home working and therefore unable to participate. Because our results suggest differential accuracy of the estimation methods, particularly for the Kawasaki and INTERSALT methods, future research should include larger sample sizes of men. In addition, the concentration of urinary sodium varied within-person between the initial and follow-up collection, as did the volume of the 24-h urine sample. In our study, the morning fasting urine collection was more convenient for participants and the investigators, but some studies suggested that the sodium concentration in afternoon or evening spot specimens better represents 24-h urinary sodium excretion [32, 33]. Last, although 24-h sodium excretion is the recommended method to assess population mean sodium intake, the evaluation of sodium intakes by 24-h urinary sodium excretion using formulae from morning fasting urine might be inaccurate for individuals. This convenient measure should be used to estimate 24-h urinary sodium excretion and mean sodium/salt intakes at the population level. Some study indicated that 24-h urinary sodium excretion measurement as a “gold standard” for evaluating salt intakes might exist variability [34, 35]. However, compared to other methods for evaluating the daily salt intake for population researches, such as food frequency questionnaire method, 24-h urinary sodium excretion measurement might be a relatively accurate approach. In addition, more studies should be explored in Chinese population.

## Conclusions

Results of our analysis suggested that three estimation methods (Kawasaki, INTERSALT and Tanaka methods) underestimated measured sodium for a Chinese urban and rural population, although, compared with the other two methods, the Kawasaki method was the least biased. A more accurate method should be developed to estimate the 24-h urinary sodium excretion from spot urine for assessment of sodium intakes in the Chinese population.

## Supporting Information

S1 Dataset Analysis Dataset of the study. (SAV)

S1 Table The differences between estimated values and measured 24-h urinary sodium excretion in quartile groups (N = 116, means). (DOCX)

S2 Table The differences between estimated values and measured 24-h urinary sodium excretion in quartile groups (N = 116, means). (DOCX)

S3 Table The distribution of the relative differences between 3 estimation methods and measured 24-h urinary sodium excretion (N = 116, N(%)). (DOCX)

S4 Table The distribution of the absolute differences between 3 estimation methods and measured 24-h urinary sodium excretion (N = 116, N(%)). (DOCX)

## Abbreviations

BP: Blood Pressure
Cr: Creatinine
CVD: Cardiovascular Disease
CI: Confidence Interval
DBP: Diastolic Blood Pressure
K/K^+^: Potassium
MFU: Morning Fasting Urine
Na/Na+: Sodium
NaCl: Sodium Chloride
RCT: Randomized Controlled Trial
SBP: Systolic Blood Pressure
SE: Standard Error
SMU: Second Morning Urine
SU: Spot Urine
USE: Urinary Sodium Excretion
