# Supplementary material for: Validation and Assessment of Three Methods to Estimate 24-h Urinary Sodium Excretion from Spot Urine Samples in Chinese Adults
Source: PLoS One. 2016 Feb 19;11(2):e0149655. doi: 10.1371/journal.pone.0149655 (PMC4760739; doi:10.1371/journal.pone.0149655)
Supplement: S1 Table — (DOCX) [file pone.0149655.s002.docx]

**S1 Table.** The differences between estimated values and measured 24-h urinary sodium excretion in quartile groups (N=116, means)

| Measured 24-h value groups | Kawasaki - measured, mg/d | INTERSALT - measured,  mg/d | Tanaka - measured, mg/d |
| --- | --- | --- | --- |
| Sodium excretion below P_25_ (n=29) | 1647.7 | -271.6 | 191.9 |
| P_25_~P_75_ (n=58) | -602.7 | -2647.9 | -2178.7 |
| Sodium excretion over P_75_ (n=29) | -3404.4 | -5622.2 | -5054.7 |

Values are mean of the differences, computed by each estimated value minus the measured value of 24-h urinary sodium excretion. Percentile group was defined by the percentile distribution of the measured 24-h values. P < 0.001 between groups statistically.
