# Supplementary material for: Validation and Assessment of Three Methods to Estimate 24-h Urinary Sodium Excretion from Spot Urine Samples in Chinese Adults
Source: PLoS One. 2016 Feb 19;11(2):e0149655. doi: 10.1371/journal.pone.0149655 (PMC4760739; doi:10.1371/journal.pone.0149655)
Supplement: S2 Table — (DOCX) [file pone.0149655.s003.docx]

**S2 Table.** The differences between estimated values and measured 24-h urinary sodium excretion in quartile groups (N=116, means)

| 24-h urine volume groups | Kawasaki - measured, mg/d | INTERSALT - measured, mg/d | Tanaka - measured, mg/d | [Na^+^] in 24-h urine, mmol/L |
| --- | --- | --- | --- | --- |
| Volume below P_25_ (n=28) | 1428.5 | -399.3 | -70.3 | 187.2 |
| P_25_~P_75_ (n=59) | -850.7 | -2919.5 | -2429.3 | 159.5 |
| Volume over P_75_ (n=29) | -2610.49 | -4864.3 | -4209.9 | 126.5 |

Values are mean of the differences, computed by each estimated value minus the measured value of 24-h urinary sodium excretion. Percentile group was defined by the percentile distribution of the volume of 24-h urine. [Na^+^] represented the concentration of the sodium in 24-h urine. P < 0.001 between groups statistically.
