# Supplementary material for: Validation and Assessment of Three Methods to Estimate 24-h Urinary Sodium Excretion from Spot Urine Samples in Chinese Adults
Source: PLoS One. 2016 Feb 19;11(2):e0149655. doi: 10.1371/journal.pone.0149655 (PMC4760739; doi:10.1371/journal.pone.0149655)
Supplement: S3 Table — (DOCX) [file pone.0149655.s004.docx]

**S3 Table.** The distribution of the relative differences between 3 estimation methods and measured 24-h urinary sodium excretion (N=116, N(%))

| Relative difference groups | Kawasaki - measured | INTERSALT - measured | Tanaka - measured |
| --- | --- | --- | --- |
| Below -40% | 19 (16.4) | 62 (53.4) | 45 (38.8) |
| -39~-30% | 12 (10.3) | 17 (14.7) | 24 (20.7) |
| -29~-20% | 15 (12.9) | 19 (16.4) | 14 (12.1) |
| -19~-10% | 7 (6.0) | 3 (2.6) | 15 (12.9) |
| -9~9% | 28 (24.1) | 5 (4.3) | 8 (6.9) |
| 10~19% | 9 (7.8) | 1 (0.9) | 2 (1.7) |
| 20~29% | 7 (6.0) | 2 (1.7) | - |
| 30~39% | 6 (5.2) | 2 (1.7) | 1 (0.9) |
| Over 40% | 13 (11.2) | 5 (4.3) | 7 (6.0) |

Values are cases number and proportion.

The relative difference = (estimated value – measured value) / measured value×100%.
