# Supplementary material for: Validation and Assessment of Three Methods to Estimate 24-h Urinary Sodium Excretion from Spot Urine Samples in Chinese Adults
Source: PLoS One. 2016 Feb 19;11(2):e0149655. doi: 10.1371/journal.pone.0149655 (PMC4760739; doi:10.1371/journal.pone.0149655)
Supplement: S4 Table — (DOCX) [file pone.0149655.s005.docx]

**S4 Table.** The distribution of the absolute differences between 3 estimation methods and measured 24-h urinary sodium excretion (N=116, N(%))

| Absolute difference groups | Kawasaki - measured | INTERSALT - measured | Tanaka - measured |
| --- | --- | --- | --- |
| Below -2000 mg / d | 32 (27.6) | 65 (56) | 63 (54.3) |
| -1999~-1200 mg / d | 15 (12.9) | 25 (21.6) | 15 (12.9) |
| -1199~-800 mg / d | 7 (6.0) | 10 (8.6) | 10 (8.6) |
| -799~-400 mg / d | 5 (4.3) | 2 (1.7) | 10 (8.6) |
| -399~399 mg / d | 19 (16.4) | 4 (3.4) | 9 (7.8) |
| 400~799 mg / d | 10 (8.6) | 4 (3.4) | 1 (0.9) |
| 800~1199 mg / d | 7 (6.0) | 2 (1.7) | 1 (0.9) |
| 1200~1999 mg / d | 10 (8.6) | 1 (0.9) | 3 (2.6) |
| Over 2000 mg / d | 11 (9.5) | 3 (2.6) | 4 (3.4) |

Values are cases number and proportion.

The absolute difference = estimated value – measured value.

1017.39 mg NaCl, about 1 g salt, contains about 400 mg sodium (Na^+^), (400 × 58.5 / 23).
